# 1985. A Prospective, Randomized Trial to Assess a Protease Inhibitor–based Regimen Switch Strategy to Manage Integrase Inhibitor–related Weight Gain

**DOI:** 10.1093/ofid/ofad500.112

**Published:** 2023-11-27

**Authors:** William R Short, Moti Ramgopal, Debbie P Hagins, Johnnie Lee, Richard Bruce Simonson, Tien-Huei Hsu, Ping Xu, David Anderson

**Affiliations:** University of Pennsylvania, PA; Midway Specialty Care Center, Fort Pierce, Florida; Georgia Department of Public Health, Coastal Health District, Chatham CARE Center, Savannah, GA, USA, Savannah, Georgia; Janssen Scientific Affairs, LLC, Titusville, New Jersey; Janssen Scientific Affairs, LLC, Titusville, New Jersey; Janssen Scientific Affairs, LLC, Titusville, New Jersey; Janssen Research & Development, LLC, Titusville, New Jersey; Janssen Scientific Affairs, LLC, Titusville, New Jersey

## Abstract

**Background:**

Integrase inhibitor (INI)–based antiretroviral (ARV) therapies are associated with greater weight gain than non-nucleoside reverse transcriptase inhibitor– or boosted protease inhibitor–based regimens, disproportionately affecting Black and Hispanic individuals and women. The mechanisms underlying this weight gain are unknown, and there are no prospective, randomized data exploring the impact of switching ARV classes to mitigate or reverse ARV-related weight gain.

**Methods:**

DEFINE (ClinicalTrials.gov: NCT04442737) is a randomized (1:1), prospective, 48-week, active-controlled, open-label, multicenter phase 4 study evaluating switching to darunavir/cobicistat/emtricitabine/tenofovir alafenamide (D/C/F/TAF) versus continuing INI+TAF/emtricitabine (FTC) in virologically-suppressed HIV-1–infected adults who had ≥10% weight gain while on the INI-based regimen. The primary objective was to assess percent change in body weight from baseline to Week 24 in both arms. The primary endpoint was analyzed using a mixed model for repeated measures in the intent-to-treat set of randomized participants who had received ≥1 dose of study drug. Secondary endpoints included change in body mass index (BMI), waist circumference (WC), efficacy, and safety. Data through Week 24 are reported.

**Results:**

Overall, 103 adults were randomized to D/C/F/TAF (n=53) or continued INI+TAF/FTC (n=50); 30% were female and 61% were Black/African American, with median 27.0 months virologic suppression on INI+TAF/FTC (**Table 1**). Discontinuation rates were low and similar between arms. At Week 24, there was no significant difference in percent change in body weight from baseline between the D/C/F/TAF and INI+TAF/FTC arms (**Figure**). Most participants in each arm had body weight changes of ≤±3% and remained within baseline BMI and WC categories. Percent body weight changes for key subgroups are shown in **Table 2**. Switching to D/C/F/TAF was safe and well tolerated, and efficacy was maintained.

**Table 1.**
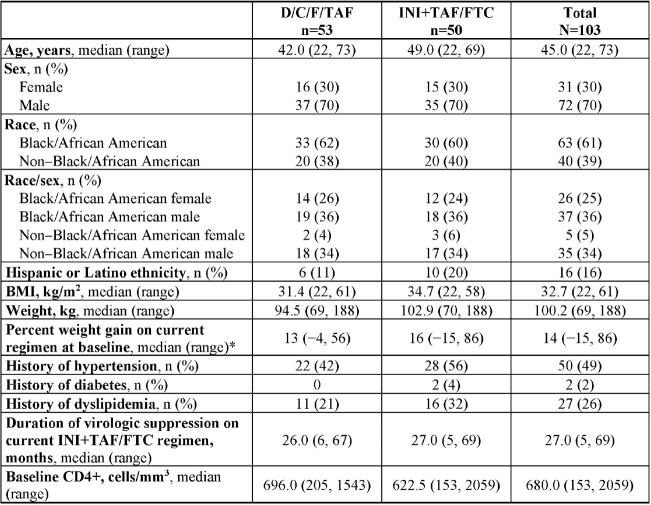
Demographics and Baseline Characteristics (ITT Set) BMI, body mass index; D/C/F/TAF, darunavir/cobicistat/emtricitabine/tenofovir alafenamide; FTC, emtricitabine; INI, integrase inhibitor; ITT, intent-to-treat; TAF, tenofovir alafenamide. *One participant in the D/C/F/TAF arm did not have available data.

Percent Change From Baseline in Body Weight Over Time for Participants Who Switched to D/C/F/TAF and Those Who Continued Their Current INI+TAF/FTC Regimen (ITT Set)

**Figure:**
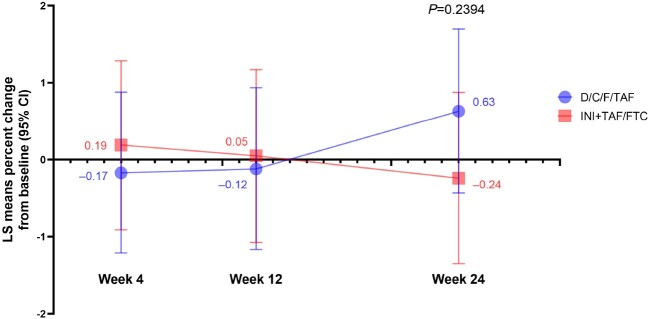

D/C/F/TAF, darunavir/cobicistat/emtricitabine/tenofovir alafenamide; FTC, emtricitabine; INI, integrase inhibitor; ITT, intent-to-treat; LS, least-squares; TAF, tenofovir alafenamide. LS means percent changes in body weight were calculated in the ITT set of randomized participants who had received ≥1 dose of study drug using a mixed model for repeated measures, in which the dependent variable was percent change from baseline in body weight; independent variables were treatment, baseline body mass index, sex, and race (Black/African American vs non–Black/African American); and visits were repeated measures. Participants in the ITT set with baseline records and ≥1 postbaseline record were included.

**Conclusion:**

There was no significant difference in weight change through 24 weeks after switching from an INI-based regimen to D/C/F/TAF in adults with INI-related weight gain. Additional analyses are ongoing, including follow up through Week 48 and evaluation of changes in biomarkers and body composition (DEXA).

**Table 2.**
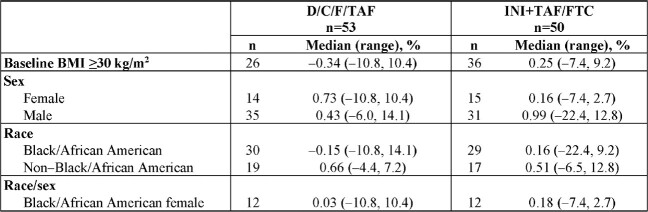
Percent Change From Baseline in Body Weight at Week 24 Among Key Subgroups (ITT Set) BMI, body mass index; D/C/F/TAF, darunavir/cobicistat/emtricitabine/tenofovir alafenamide; FTC, emtricitabine; INI, integrase inhibitor; ITT, intent-to-treat; TAF, tenofovir alafenamide.

**Disclosures:**

**William R. Short, MD**, Gilead Sciences: Advisor/Consultant|ViiV: Advisor/Consultant|ViiV: Honoraria **Moti Ramgopal, MD, FACP, FIDSA**, AbbVie: Honoraria|Gilead Sciences, Inc.: Advisor/Consultant|Gilead Sciences, Inc.: Honoraria|Janssen Pharmaceuticals: Advisor/Consultant|Janssen Pharmaceuticals: Honoraria|Merck: Advisor/Consultant|ViiV Healthcare: Advisor/Consultant|ViiV Healthcare: Honoraria **Debbie P. Hagins, MD, FAPCR, AAHIVS**, Janssen Pharmaceuticals: Advisor/Consultant **Johnnie Lee, MD**, Janssen Pharmaceuticals: Employee|Janssen Pharmaceuticals: Stocks/Bonds **Richard Bruce Simonson, BS**, Janssen Pharmaceuticals: Employee|Janssen Pharmaceuticals: Stocks/Bonds **Tien-Huei Hsu, PhD**, Janssen Pharmaceuticals: Employee|Janssen Pharmaceuticals: Stocks/Bonds **Ping Xu, PhD**, Janssen Pharmaceuticals: Employee|Janssen Pharmaceuticals: Stocks/Bonds **David Anderson, MD**, Janssen Pharmaceuticals: Employee|Janssen Pharmaceuticals: Stocks/Bonds

